# Case Report: Clinical and molecular genetic analysis of a patient with coexisting complete androgen insensitivity syndrome and neurofibromatosis type 1 and 15pstk + polymorphism

**DOI:** 10.3389/fped.2026.1919358

**Published:** 2026-08-26

**Authors:** Wei Wang, Yake Jiao, Yang Xiu, Jing Wang, Yanyan Hu

**Affiliations:** 1 Linyi People's Hospital, Shandong Second Medical University, Linyi, Shandong Province, China; 2 Department of Children's Health, Linyi People's Hospital, Linyi, Shandong Province, China; 3 Binzhou Medical University, Yantai, Shandong Province, China; 4 Fei County People's Hospital, Linyi, Shandong Province, China; 5 Jinzhou Medical University, Jinzhou, Liaoning Province, China

**Keywords:** androgen insensitivity syndrome, comorbidity, disorders of sex development, genetic variation, neurofibromatosis 1

## Abstract

**Purpose:**

To report a pediatric patient with coexisting complete androgen insensitivity syndrome (CAIS), neurofibromatosis type 1 (NF1), and 15pstk + polymorphism, and to analyze its clinical phenotypes and molecular genetic characteristics.

**Methods:**

Clinical data of a 7-year-and-11-month-old patient with female social gender were retrospectively analyzed. Pathogenic gene variants were identified by whole-exome sequencing (WES), pedigree verification was performed by Sanger sequencing, and variant pathogenicity was evaluated using bioinformatics tools.

**Results:**

The patient exhibited typical phenotypes of both diseases, accompanied by unique features, including epicanthal folds, webbed neck, broad great toes and thumbs. WES identified a maternally inherited hemizygous missense variant in the *AR* gene (c.2599G > A, p.Val867Met) and a *de novo* heterozygous missense variant in the *NF1* gene (c.5488C > T, p.Arg1830Cys).

**Conclusion:**

This is the first report of concurrent CAIS and NF1, which enriches and expands the genotypic and phenotypic spectra of both disorders.

## Introduction

Androgen insensitivity syndrome (AIS) is an X-linked recessive disorder of sex development (DSD). Pathogenic variants in the *AR* gene located on the long arm of the X chromosome (Xq11–q12) impair the responsiveness of target tissues to androgens, leading to failure of androgen signaling to exert normal biological effects in peripheral tissues. According to the degree of feminization, AIS is categorized into complete androgen insensitivity syndrome (CAIS), partial androgen insensitivity syndrome (PAIS), and mild androgen insensitivity syndrome (MAIS). The incidence of CAIS ranges from 1/20,400 to 1/99,000. Owing to the secretion of anti-Müllerian hormone by testicular Sertoli cells, patients with CAIS lack the uterus, cervix, and proximal vagina. The classic clinical manifestations include primary amenorrhea at puberty or inguinal swelling during infancy (1).

Neurofibromatosis type 1 (NF1) is an autosomal dominant disorder with an estimated prevalence of 1 in 2,000 to 4,000 individuals, caused by loss-of-function variants in the *NF1* gene mapped to 17q11.2. This gene encodes neurofibromin, a 2,818-amino-acid protein that catalyzes the hydrolysis of active RAS-GTP to inactive RAS-GDP, thereby serving as a negative regulator of the RAS signaling pathway (2, 3). The disease generally manifests in childhood and presents with highly variable phenotypes, characterized by café-au-lait macules, axillary and/or inguinal freckling, Lisch nodules (iris hamartomas), neurofibromas, optic pathway gliomas and distinctive skeletal abnormalities (4).

CAIS and NF1 are caused by pathogenic variants in the *AR* and *NF1* genes respectively, with distinct causative genes, molecular pathways and inheritance patterns. In the present study, the clinical phenotypes and molecular genetic features of the patient were comprehensively analyzed to elaborate the clinical implications of the co-occurrence of these two rare genetic variants.

## Case report

A 7-year-and-11-month-old female patient with a karyotype of 46,XY,15pstk + was enrolled in this study. She presented to our hospital for definitive diagnosis, following a bilateral inguinal hernia repair performed 2 years prior, during which no uterus or ovaries were identified intraoperatively. The patient was the elder of dizygotic twins, delivered by cesarean section at full term with a birth weight of 3.15 kg. She achieved independent walking and verbal speech at the age of 1 year and 4 months. Her parents and the younger dizygotic twin sister were healthy, with no relevant family history of genital anomalies, endocrine disorders, or genetic diseases.

## Materials and methods

### Laboratory examinations

Serum levels of luteinizing hormone (LH), follicle-stimulating hormone (FSH), estradiol (E2), testosterone (T), prolactin (PRL), progesterone (P), alpha-fetoprotein (AFP), anti-Müllerian hormone (AMH), free triiodothyronine (FT3), free thyroxine (FT4), thyroid-stimulating hormone (TSH), 17-hydroxyprogesterone (17-OHP), adrenocorticotropic hormone (ACTH), and cortisol (COR) were measured using a fully automated chemiluminescence immunoassay system (Siemens Advia Centaur XP, Siemens Healthineers, Germany). Liver and kidney function and electrolyte profiles were analyzed using a fully automated biochemical analyzer (Cobas c702, Roche Diagnostics, Switzerland).

### Human chorionic gonadotropin (hCG) stimulation test

A single intramuscular injection of recombinant hCG (1,000 IU) was administered; serum testosterone concentrations were retested 3 days after injection. Result interpretation adhered to the UK consensus for DSD evaluation: a normal hormonal response is defined as post-stimulation testosterone either exceeding the upper bound of the prepubertal reference range or rising ≥2-fold above baseline; such adequate testosterone elevation confirms functionally active testicular tissue with intact steroidogenic capacity. A blunted response (failure to meet either threshold) indicates impaired Leydig-cell function or endogenous defects in the testosterone biosynthetic pathway (5).

### Ultrasound examination

Ultrasonography was performed using a Logic E9 ultrasound system (GE Healthcare, USA) equipped with a linear-array probe (8–12 MHz) to conduct high-frequency grayscale and color Doppler scanning of both inguinal regions. The examination aimed to locate and describe the echotexture, dimensions, and blood flow characteristics of any palpable masses or suspected gonadal tissue.

### Ophthalmic assessment

A comprehensive ophthalmic examination was conducted to screen for Lisch nodules, a diagnostic feature of NF1. This included slit-lamp biomicroscopy (Haag-Streit BQ 900, Switzerland) under mydriasis. Additionally, fundus imaging was performed using spectral-domain optical coherence tomography (Spectralis, Heidelberg Engineering, Germany).

### Cytogenetic analysis

Conventional karyotyping was performed on phytohemagglutinin-stimulated peripheral blood lymphocytes using G-banding techniques at a resolution of approximately 550 bands per haploid genome. Chromosome analysis followed the guidelines of the International System for Human Cytogenomic Nomenclature (ISCN 2020).

### Whole-Exome sequencing (WES)

Genomic DNA was extracted from the patient's peripheral blood using a commercial kit (Bosheng Medical Laboratory, Hangzhou, China). The extracted DNA was fragmented, and a sequencing library was constructed through end repair, adenylation, and adapter ligation. Human exonic regions were captured using the xGen® Exome Research Panel v2.0. Following capture, the enriched DNA was eluted, amplified, purified, and subjected to high-throughput sequencing on an Illumina NovaSeq 6,000 platform. The generated sequences were aligned to the hg19 human reference genome. Genetic variants were identified using dedicated analysis software, which also provided metrics for target region coverage and average sequencing depth (mean coverage >99%). Subsequently, variants were annotated for nucleotide/amino acid conservation, predicted biological function, and population frequency (e.g., gnomAD). They were then cross-referenced with entries in public databases, including Online Mendelian Inheritance in Man (OMIM), the Human Gene Mutation Database (HGMD), and ClinVar.

### Sanger sequencing validation and segregation analysis

Candidate genes identified by whole-exome sequencing were validated in the proband via Sanger sequencing, and subsequent segregation analysis was performed in the proband's parents and dizygotic twin sister to further confirm their inheritance pattern. For this purpose, specific primer pairs were designed to flank each variant locus: for the *NF1* c.5488C > T variant (reference sequence NM_000267.3), primers NF1-F (5'-GCTGCTGCTGCTGCTGCTG-3’) and NF1-R (5'-CAGCTGCTGCTGCTGCTGTA-3’) were used; for the *AR* c.2599G > A variant (reference sequence NM_000044.6), primers AR-F (5'-AGCACACAGACTTCAACTAACAGG-3’) and AR-R (5'-GACCCGTGTTCTTTTCTTCACCAC-3’) were used. PCR amplification was carried out using the KAPA2G Robust HotStart PCR Kit (Roche), and the resulting products were purified prior to sequencing. The interpretation of all sequence variants adhered to the standards and guidelines established by the American College of Medical Genetics and Genomics (ACMG).

### Bioinformatic analysis

MUpro web server (http://mupro.proteomics.ics.uci.edu/) was used to predict the impact of AR gene variants on protein stability. The three-dimensional (3D) structural models of the wild-type and mutant AR proteins were constructed using SWISS-MODEL (https://swissmodel.expasy.org/interactive). PyMOL 2.5 software was employed for visualization and analysis of the effects of variants on protein structure. Amino acid sequences of AR proteins across different species were retrieved from the UniProt database (http://www.uniprot.org/), and Clustal X software was used to analyze the cross-species conservation of the variant amino acids. Additionally, PolyPhen-2 (genetics.bwh.harvard.edu/pph2/) and MutationTaster (http://www.mutationtaster.org/) were applied to predict the pathogenicity of AR gene variants.

## Results

### Clinical features

**Figure 1:**
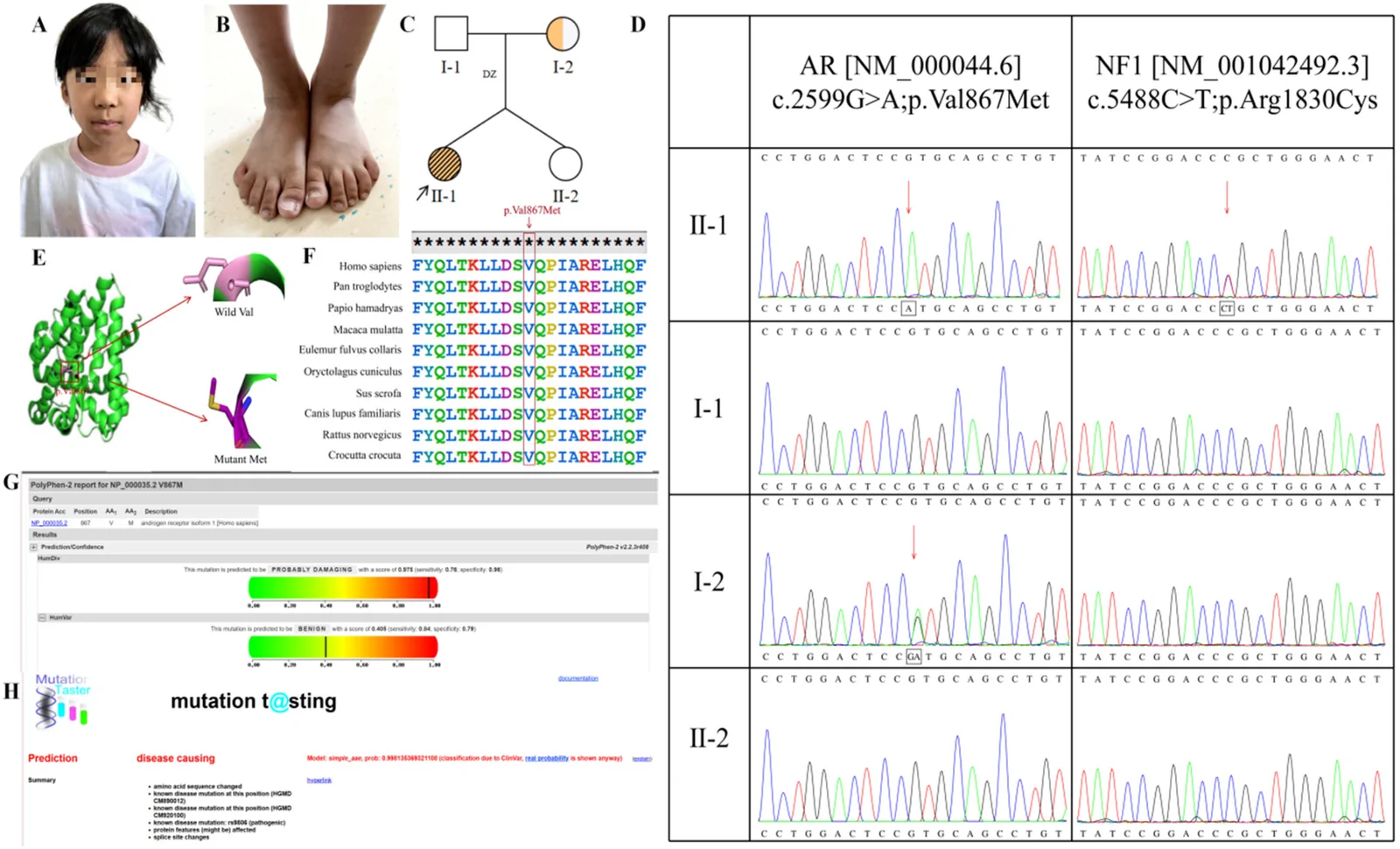
Clinical manifestations and genetic analyses. **(A)** Multiple scattered café-au-lait macules on the lower extremities and broad great toes. **(B)** Multiple scattered café-au-lait macules on the neck and webbed neck. **(C)** Pedigree chart. I-1: Father, normal male; I-2: Mother, carrier of the *AR gene* mutation without phenotypic expression; II-1: Proband (indicated by the arrow), carrying the *AR gene* variant and a *de novo* *NF1 gene* variant, with phenotypes consistent with the clinical diagnosis of AIS complicated with NF1; II-2: Dizygotic twin sister, normal female. DZ: Dizygotic twins. **(D)** Pedigree Sanger sequencing results: Proband II-1 carried the *AR gene* variant c.2599G > A (p.Val867Met), confirmed to be maternally inherited; the *NF1 gene* variant c.5488C > T (p.Arg1830Cys) was a *de novo* mutation. **(E)** Superimposed 3D structural models of wild-type and mutant AR proteins around residue 867. **(F)** Multi-species sequence alignment showing high conservation of valine at position 867. **(G)** PolyPhen-2 prediction result: Probably damaging. **(H)** MutationTaster prediction result: Disease-causing.

The patient measured 120.4 cm in height (−1.41 SDS) with a target parental height of 161.5 cm, weighed 23 kg, and had a body mass index of 15.9 kg/m^2^. Physical examination revealed scattered café-au-lait macules over the skin, accompanied by epicanthal folds, webbed neck, broad thumbs and toes (Figures 1A,B). Female external genitalia were present with mild clitoral hypertrophy, partial fusion of the labia minora, and hypoplasia of the labia majora. External genitalia were graded as Prader stage 2. The anogenital distance measured 2 cm, the external masculinization score (EMS) was 2, corresponding to Tanner stage 1, with bilateral palpable inguinal masses identified on examination. Two linear surgical scars were visualized on the abdomen. Neurological examination revealed no abnormalities.

Her younger dizygotic twin sister (7 years 11 months) showed no abnormalities on physical examination and exhibited no signs of NF1 or CAIS.

Laboratory test results showed the child's baseline testosterone was lower than the normal reference level, AMH was markedly elevated, and FSH showed a mild increase. After hCG stimulation, testosterone rose prominently to 696.820 ng/dL, and this pronounced elevation indicates the gonadal tissue preserves intact testosterone-synthesizing function and confirms the existence of functional ectopic testicular tissue. Thyroid function, hepatic function, lipid profile, serum tumor markers and routine hematological parameters were all within normal limits. Full tabulated laboratory data with reference intervals are available in the Supplementary Materials.

High-frequency ultrasound revealed bilateral hyperechoic nodules with well-defined borders and homogeneous internal echoes in the inguinal regions, morphologically consistent with ectopic testicular tissue. Ophthalmologic examination, including dilated slit-lamp microscopy and spectral-domain optical coherence tomography, showed no evidence of Lisch nodules or optic pathway gliomas. Conventional G-banding karyotyping yielded the result 46,XY,15pstk+, indicating chromosomal polymorphism with a 15p stalk extension.

### Whole-exome sequencing (WES)

Whole-exome sequencing (WES) identified a hemizygous missense variant in the AR gene (NM_000044.6: c.2599G > A, p.Val867Met) in the proband. This variant results in the substitution of valine with methionine at amino acid position 867.Additionally, WES detected a heterozygous missense variant in the NF1 gene (NM_001042492.3: c.5488C > T, p.Arg1830Cys). Sanger validation of the parental samples confirmed it arose as a *de novo* mutation exclusive to the proband, with neither parent carrying this alteration.We conducted a systematic literature and database search for the two variants identified in this case. The *AR* gene variant c.2599G > A (p.Val867Met) resides within the ligand-binding domain. It is classified as pathogenic in ClinVar and represents a well-established pathogenic variant underlying androgen insensitivity syndrome. Functional studies have verified that this variant impairs DHT-dependent transcriptional activation of the androgen receptor (6).The *NF1* variant c.5488C > T (p.Arg1830Cys) is also designated pathogenic in ClinVar. Notably, this variant corresponds to p.Arg1809Cys when annotated against the alternative canonical transcript NM_000267.4. Published literature indicates this variant is generally associated with a mild NF1 phenotype characterized by abundant café-au-lait macules but infrequent neurofibromas (7). To date, no standalone functional research specifically investigating p.Arg1830Cys has been published.

### Sanger sequencing validation and segregation analysis

Sanger sequencing confirmed the presence of the *AR* c.2599G > A (p.Val867Met) variant in the proband. Segregation analysis of the family revealed that this variant was inherited from the proband's mother, who is a heterozygous carrier. The variant was not detected in the proband's father or her dizygotic twin sister. For the *NF1* c.5488C > T (p.Arg1830Cys) variant, Sanger sequencing confirmed its presence in the proband and demonstrated that it is a *de novo* variant, with no detection in the proband's parents or twin sister (Figures 1C,D).

### Bioinformatic analysis

MUpro predicted that the *AR* c.2599G > A (p.Val867Met) variant reduces protein stability with a *ΔΔ*G value of −1.1227616. Structural superimposition revealed severe steric clashes specifically at residue 867, arising from the elongated thioether side chain of mutant methionine (Figure 1E). This side chain creates steric hindrance within the androgen ligand-binding pocket, breaks native hydrophobic stacking at this site, destabilizes the local conformation of the ligand-binding domain, and ultimately weakens androgen-receptor binding affinity. Importantly, the global folding backbone of the androgen receptor stays unaltered by this single substitution. Cross-species alignment confirmed high evolutionary conservation of amino acid position 867 (Figure 1F). PolyPhen-2 and MutationTaster further predicted this variant to be possibly damaging and disease-causing, respectively (Figures 1G,H).

### Variant pathogenicity classification

The two variants were interpreted and classified according to the ACMG/AMP guidelines. For the *AR* c.2599G > A (p.Val867Met) variant located on the X chromosome, the combined evidence (PS3_P, PS4_M, PM2_P, PP3, PP4) supports a likely pathogenic classification. This hemizygous variant is sufficient to account for the patient's clinical phenotype. In contrast, the *de novo* heterozygous *NF1* c.5488C > T (p.Arg1830Cys) variant was assessed as pathogenic based on a more robust combination of evidence (PS2, PS4, PM2_P, PP2, PP3_M).

## Discussion

This report presents an exceedingly rare case of co-occurrence of complete androgen insensitivity syndrome (CAIS) and neurofibromatosis type 1 (NF1). An extensive literature search revealed documented instances of NF1 co-occurring with other conditions, such as X-linked ichthyosis (8) and nevoid basal cell carcinoma syndrome (9), as well as reports of CAIS co-occurrence with other disorders like Klinefelter syndrome (10), and no prior records of such a case have been found in domestic or international literature or databases.

The height SDS of the patient in this case was −1.41. Both CAIS and NF1 exert regulatory effects on body height. Previous literature has documented that adult patients with CAIS generally attain a final height higher than the average female height yet lower than the average male height, approximately 165.7 cm (11). Studies have verified that children with NF1 present markedly shorter stature compared with the general population; offspring affected by NF1 born to unaffected parents have a body height inferior to the target midparental height as well as the height of their unaffected siblings (12). To date, no published cohort or case studies have specifically explored growth patterns in individuals complicated with both CAIS and NF1. Accordingly, this study only objectively describes the respective growth regulatory characteristics of the two disorders.

The patient exhibited epicanthal folds, webbed neck, broad thumbs and broad great toes, none of which represent typical manifestations of either CAIS or classic NF1. Noonan syndrome is characterized by cardinal features, including webbed neck, hypertelorism, distinctive facial dysmorphism and short stature, many of which overlap with the physical signs observed in this child. Nevertheless, whole-exome sequencing failed to identify pathogenic variants in *PTPN11, SOS1, RAF1, KRAS* and other causative genes, thereby ruling out the diagnosis of Noonan syndrome (13). Large segmental microdeletions encompassing the *NF1* gene at 17q11.2 constitute a special subtype of NF1, which is frequently accompanied by craniofacial malformations such as epicanthal folds and down-slanting palpebral fissures. The underlying mechanism involves persistent overactivation of the RAS-MAPK pathway secondary to loss of NF1 function, which disrupts the embryonic differentiation of neural crest cells and mesenchymal tissues (14). Flanking microdeletions at 17q11.2 that spare the coding region of NF1 can also elicit comparable phenotypic presentations (15), yet no such copy number variations were detected in the present case. Collectively, these atypical manifestations may stem from the inherent phenotypic variability of NF1, isolated benign congenital variations, or occult genetic alterations that cannot be captured by whole-exome sequencing.

The karyotype 46, XY, 15pstk + detected in this case represents a structural heteromorphism. According to the International System of Nomenclature of Human Cytogenetics (ISCN) 2024 edition, this karyotype specifically denotes an elongation of the short arm of chromosome 15 along the stalk, classified as a clinically common benign chromosomal variation (16). Its molecular basis primarily involves copy number variation in the tandemly repeated ribosomal DNA (rDNA) sequences located within the nucleolar organizer region (NOR) at 15p12 (17). Chromosomal stalk-length variants (including stalk elongation) on D-group chromosomes are detected in approximately 2%–5% of healthy populations, with minor discrepancies across cohorts due to sample size and ethnic differences. As a frequent benign variant, 15pstk + carries no functional gene defects and requires no targeted clinical intervention (18, 19). It is regarded as an incidental benign genetic background variant.

Standardized diagnosis and treatment guidelines for the rare co-occurrence of CAIS and NF1 are currently unavailable; hence, individualized clinical evaluation should be conducted by a multidisciplinary team (MDT). For patients with isolated CAIS, gonadectomy is generally recommended after puberty. This timing facilitates the development of feminine secondary sexual characteristics and optimal skeletal maturation, while lowering the risk of malignant transformation of gonadal tissue. Estrogen replacement therapy (ERT) should be initiated promptly after surgery to induce and sustain female secondary sexual traits, as well as preserve skeletal and cardiovascular health (20). The absence of neurofibromin raises the level of GTP-bound Ras, enhances the activation of the downstream RAS/MAPK signaling pathway, and further increases the risk of malignant transformation in various tissues (21). Although the risk of testicular malignant tumors is extremely low (< 1%) in prepubertal patients with isolated CAIS (22), the timing of gonadectomy still needs to be reassessed in this case. Meanwhile, NF1-deficient cells are highly sensitive to estrogen; an estrogen-rich microenvironment synergizes with excessive activation of the RAS pathway to facilitate tumor cell proliferation and malignant progression (23). Therefore, estrogen replacement therapy (ERT) should be administered with great caution. Clinically, a regimen starting with a low dose and gradual dose titration is advised to maintain the minimum effective serum estrogen concentration required for physical development. This regimen can meet physiological developmental needs while minimizing the risk of estrogen-mediated tumor progression.

This study has several limitations. First, it is a retrospective single-case report, which prevents definitive conclusions on the genotype–phenotype correlation of the identified NF1 variant. Second, the patient's mother did not receive karyotyping, endocrine testing or X-chromosome inactivation analysis, so we cannot confirm her carrier status or trace the origin of aberrant chromosomal variants. Third, gonadal histopathology and *in vitro* androgen receptor functional assays were not performed, restricting precise evaluation of gonadal morphology and mutant receptor activity. Fourth, key clinical data including head circumference, developmental milestones and cognitive assessment were not collected, limiting comprehensive phenotypic analysis of NF1-related features. In addition, only cross-sectional data are available with no long-term follow-up records to evaluate pubertal development and long-term management outcomes. Further multi-case studies and prolonged follow-up are required to establish standardized individualized management strategies for patients with concurrent CAIS and NF1.

This case report describes the first coincidental case complicated by both CAIS and NF1, which are separately induced by pathogenic variants in the independent *AR* and *NF1* genes. The simultaneous occurrence of these two monogenic disorders is extremely rare, and the presence of multiple atypical clinical manifestations posed considerable challenges for differential diagnosis. Based on this case, we highlight that children presenting with complex or atypical disorders of sex development should be screened for coexisting additional diseases, and comprehensive molecular genetic testing ought to be performed.

## Data Availability

The original contributions presented in the study are included in the article/Supplementary Material, further inquiries can be directed to the corresponding author.
