# Supplementary material for: Case Report: Clinical and molecular genetic analysis of a patient with coexisting complete androgen insensitivity syndrome and neurofibromatosis type 1 and 15pstk + polymorphism
Source: Front Pediatr. 2026 Aug 26;14:1919358. doi: 10.3389/fped.2026.1919358 (PMC13553875; doi:10.3389/fped.2026.1919358)
Supplement: Supplementary file 1 [file Table1.docx]

****Full Hormone and Biochemical Test Data****

****Detection Methods****

**Serum luteinizing hormone (LH), follicle‑stimulating hormone (FSH), estradiol (E₂), testosterone (T), prolactin (PRL), progesterone (P), alpha‑fetoprotein (AFP), anti‑Müllerian hormone (AMH), free triiodothyronine (FT₃), free thyroxine (FT₄), thyroid‑stimulating hormone (TSH), 17‑hydroxyprogesterone (17‑OHP), adrenocorticotropic hormone (ACTH) and cortisol (COR) were measured by fully automated chemiluminescence immunoassay (Siemens Advia Centaur XP, Siemens Healthineers, Germany). Hepatorenal function and electrolytes were analyzed using a fully automated biochemical analyzer (Cobas c702, Roche Diagnostics, Switzerland).**

****Reference Intervals****

**LH: 1.9–12.5 mIU/mL; FSH: 2.5–10.2 mIU/mL; E₂: 19.5–144.2 pg/mL; T: 8.38–35.01 ng/dL; PRL: 2.8–29.2 ng/mL; P: 0–1.40 ng/mL; AFP: 0–7 ng/mL; AMH: 0.15–16.0 ng/mL; FT₃: 3.5–6.5 pmol/L; FT₄: 11.5–22.7 pmol/L; TSH: 0.64–6.27 μIU/L; 17‑OHP: 0–2.28 ng/mL; ACTH: 0–46 pg/mL; morning cortisol: 5–25 μg/dL.**

****Patient Laboratory Results****

**Baseline hormone indices: LH 0.42 mIU/mL, FSH 12.80 mIU/mL, E₂ 36.06 pg/mL, T <7.00 ng/dL, PRL 5.06 ng/mL, P 0.25 ng/mL, AFP 1.18 ng/mL, AMH >22.9 ng/mL, FT₃ 6.08 pmol/L, FT₄ 16.09 pmol/L, TSH 4.197 μIU/L, 17‑OHP 0.373 ng/mL, ACTH 17.2 pg/mL, 8‑a.m. cortisol 3.36 μg/dL.**

**Post‑stimulation result of the hCG test: T 696.820 ng/dL.**
